# Phosphorus-Deficiency-Induced Development of Root Apoplastic Barriers Restricts Cadmium Translocation in *Salix caprea*

**DOI:** 10.3390/plants15111728

**Published:** 2026-06-03

**Authors:** Ao Li, Yongge Wang, Yuxiao Qu, Junzhu Zou, Guansheng Ju, Zhenyuan Sun, Junxiang Liu

**Affiliations:** 1 Research Institute of Forestry, Chinese Academy of Forestry, Beijing 100091, China; liao@caf.ac.cn (A.L.);; 2 Beijing Academy of Forestry and Landscape Architecture, Beijing 100102, China

**Keywords:** casparian strip, heavy metal, phosphorus, *Salix caprea*, suberin lamellae

## Abstract

Phosphorus (P) plays a crucial role in the translocation and accumulation of cadmium (Cd) in plants; however, its effects on Cd transport via the apoplastic pathway remain unclear. In this study, *Salix caprea* was used to systematically investigate the regulatory roles of P on apoplastic barrier deposition (casparian strips and suberin lamellae), apoplastic Cd transport, and Cd accumulation through an integrated approach combining physiological, biochemical, anatomical, and transcriptomic analyses. The results showed that under Cd stress, P-deficient conditions accelerated the development of apoplastic barriers, with the initiation of casparian strips and suberin lamellae occurring 0.5% and 5% closer to the root tip, respectively, compared with P-sufficient conditions. Transmission electron microscopy (TEM) further revealed that P deficiency significantly increased the thickness of endodermal cell walls by 37.2% relative to P sufficiency when exposed to Cd stress. Moreover, root lignin content and the activities of lignin- and suberin-related enzymes (POD and PAL) were significantly higher under P deficiency. Transcriptome analysis indicated that under Cd stress, P deficiency markedly upregulated genes involved in lignin and suberin monomer biosynthesis (*PAL*, *POD*, *KCS20*, *LACS*), as well as casparian strip polymerization (*CASP*, *MYB36*). In addition, under P-deficient conditions, the net Cd^2+^ flux at the root tip was reduced by 21.3%, and the 8-hydroxy-1,3,6-pyrenetrisulfonic acid trisodium salt (PTS, a tracer for the apoplastic pathway) concentration in leaves decreased by 36.3%, further confirming that P deficiency limits Cd transport via the apoplastic route. This may explain why, under P-deficient conditions, Cd concentrations in leaves and shoots were significantly reduced by 48.7% and 63%, respectively, compared with P-sufficient conditions. This study provides new insights into improving phytoremediation efficiency for extreme heavy metal pollution by P application.

## 1. Introduction

Cadmium (Cd) is a widespread heavy metal with significant toxic effects on organisms [1]. Thus, the remediation of Cd-contaminated soils is crucial. Phytoremediation has emerged as a widely researched and applied approach due to its in situ nature and safe characteristics compared to traditional remediation methods [2]. However, the efficiency of phytoremediation largely depends on the degree of Cd enrichment in aboveground plant parts, a process heavily reliant on the radial transport of Cd from the root epidermis to the xylem [3,4]. Radial ion transport in roots typically involves three pathways: apoplastic, symplastic and coupled transcellular [5,6]. While numerous studies have suggested that Cd transport relies on the transcellular pathway facilitated by transporter proteins, recent emphasis has been placed on the significant role of the apoplastic pathway in Cd radial transport [7,8]. For instance, research has provided evidence that the apoplastic pathway can contribute significantly to Cd accumulation, with contributions reaching up to 37% [9]. Additionally, the phytoremediation process is highly susceptible to the inferior soil properties of contaminated areas, especially phosphorus (P) deficiency, which can potentially hinder the remediation process [10]. P is an indispensable inorganic nutrient element required for plants, playing an important role in various metabolic processes [11]. Numerous studies have highlighted the critical role of P in plant responses to Cd [12,13]. However, most research on Cd transport mediated by P has concentrated on transmembrane pathways related to plasma or vacuolar membranes [14,15], and there is still a gap in knowledge regarding the effects of P on Cd transport via the apoplastic pathway.

The differentiation of the plant endodermis involves two primary stages: the formation of the casparian strip (CS) and the suberin lamellae (SL) [5]. The CS formed in the first stage of differentiation is a belt-like structure, with the anticlinal primary cell walls of the endodermal cells impregnated with lignin [16]. The SL formed in the second stage of differentiation refers to the deposition of suberin between the primordial cell wall and the plasma membrane [17]. Extensive research has highlighted the plasticity of the apoplastic barriers, which includes a CS and SL. Various abiotic stresses, including heavy metal stress and nutrient stress, have been demonstrated to notably influence the development of apoplastic barriers [18,19,20,21]. In addition, stress-induced deposition of the apoplastic barriers markedly impacts substance transport through the apoplastic pathway. Enhanced apoplastic barriers have been found to substantially inhibit the transport of water and ions to the xylem [22]. Conversely, delayed deposition of apoplastic barriers favors Cd translocation [23,24,25]. Moreover, recent studies have indicated that mineral elements play a role in mediating the deposition of CSs and SL in root tips under heavy metal stress, subsequently influencing heavy metal transport processes in plants. For instance, in *Capsicum annuum*, transmission electron microscopy (TEM) and single-cell RNA sequencing have demonstrated that adequate boron (B) significantly induces CS deposition, thereby inhibiting Cd translocation [26]. In contrast, low calcium (Ca) levels induced a delay in the deposition of apoplastic barriers, leading to enhanced Cd accumulation in *Sedum alfredii* [27]. Therefore, we speculate that mineral element-mediated modifications of the root apoplastic barriers play an important role as a plant response mechanism to heavy metal stress. Despite several studies emphasizing the regulatory roles of P and Cd on apoplastic barrier development [4,28], the specific influence of P on apoplastic barrier development under Cd stress, as well as the regulation of the apoplastic barriers on Cd accumulation, is still unclear.

Willows are notable for their perennial nature and high biomass production, making them an ideal choice for phytoremediation applications. Moreover, numerous previous studies have highlighted the significant role of apoplastic barriers in Cd enrichment in willows [29]. In addition, P deficiency is commonly observed in areas affected by heavy metal pollution. Therefore, in this study, we investigated how P deficiency affects Cd accumulation by influencing the deposition of root apoplastic barriers using a pre-screened clone of *Salix caprea* with high Cd accumulation. The study encompassed the following: (1) the effects of P deficiency on Cd accumulation and translocation; (2) the effects of P deficiency on root apoplastic barrier development; and (3) the physiological and molecular mechanisms by which P deficiency regulates apoplastic barrier development.

## 2. Results

### 2.1. P Deficiency Affects the Development of CS and SL

**Figure 1:**
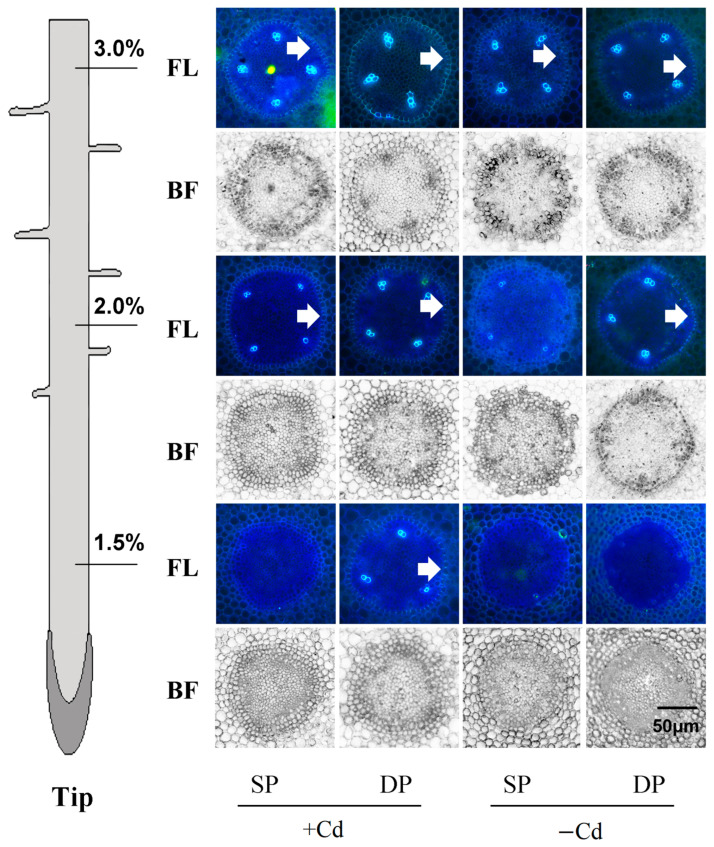
The formation of casparian strip (CS) in roots of *Salix caprea*. The scale bars represent 50 µm. The white arrows indicate the occurrence of CS. BF: bright field images. FL: fluorescence images. SP: sufficient P. DP: deficient P.

We first observed the formation of CSs in the root tips (Figure 1). The results demonstrated that CS formation was significantly affected by both Cd and P. Regardless of P treatment, Cd exposure reduced the distance from the root tip to the initiation site of CS formation. Under P deficiency, CS formation was promoted, and this effect was largely independent of Cd exposure, as indicated by the substantially shorter distance between the CS initiation site and the root tips. Among all treatments, the latest-forming CS was observed under the combination of Cd-free and sufficient P conditions, approximately 3.0% from the root tips. Conversely, CS formation occurred earliest under the combined condition of Cd exposure and P deficiency, at a distance of approximately 1.5% from the root tips. In summary, both Cd exposure and P deficiency promoted CS formation, and P deficiency further accelerated CS development under Cd stress.

**Figure 2:**
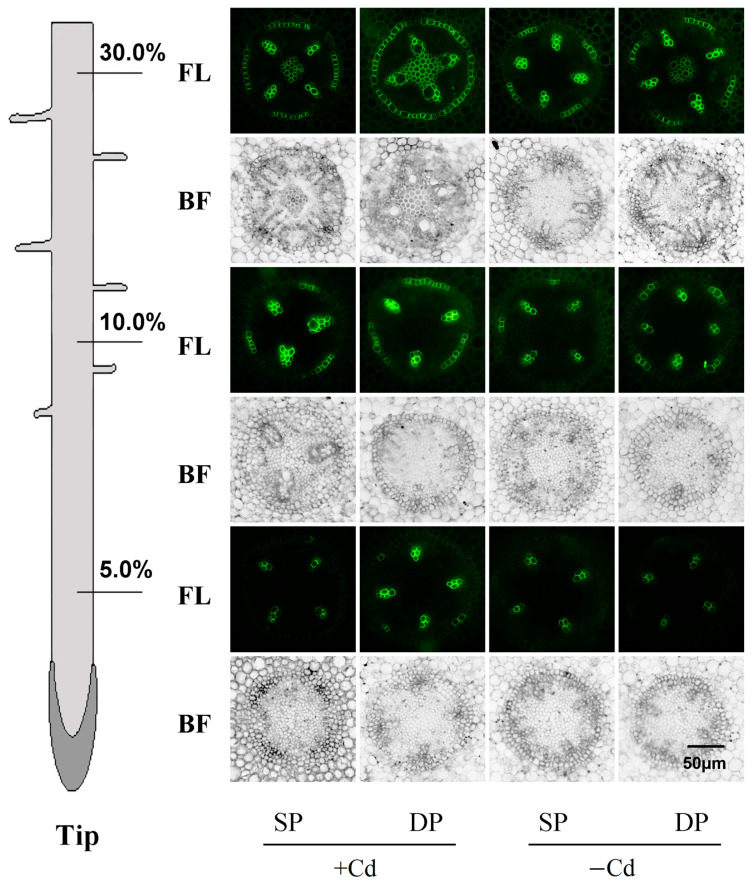
The formation of suberin lamellae (SL) in roots of *Salix caprea*. The cross-section of SL staining. The scale bar represents 50 µm. BF: bright field images, FL: fluorescence images. SP: sufficient P, DP: deficient P.

**Figure 3:**
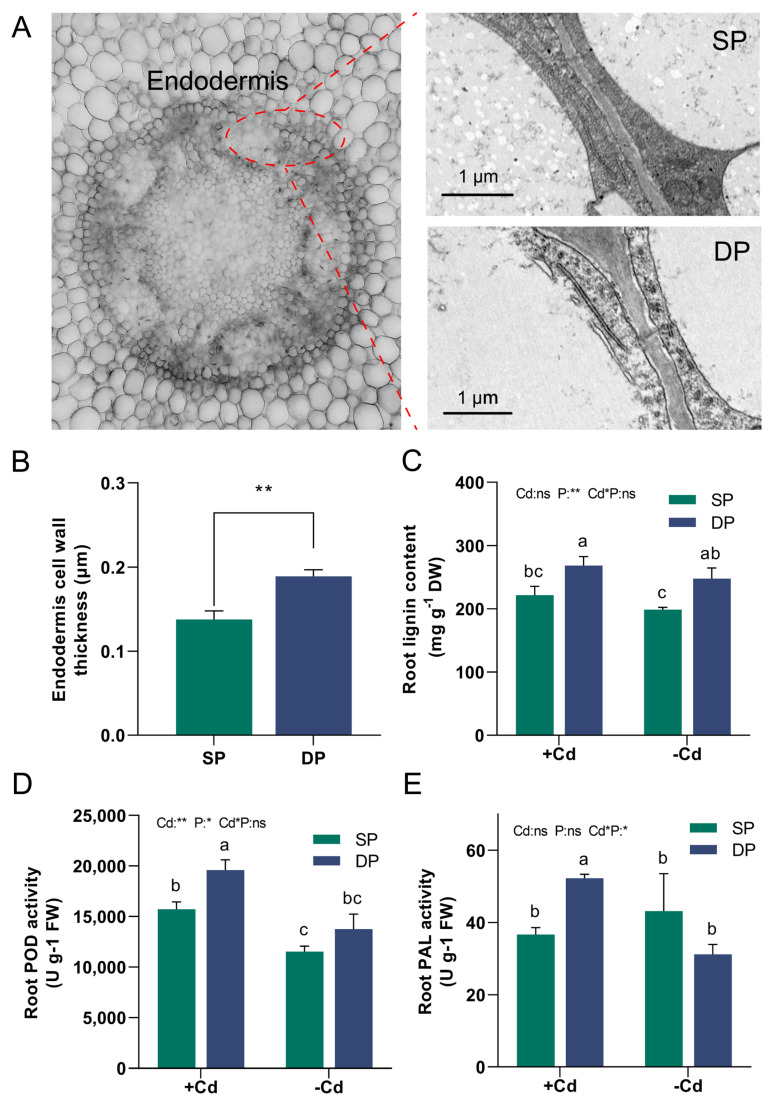
Effect of P and Cd treatments on endodermal cell wall thickness and lignin-related enzyme activities in root tips. (**A**) Effect of P on endodermal cell wall thickness in root tips under Cd stress (transmission electron microscopy analysis, scale bar represents 1 µm). (**B**) The cell wall thickness of endodermal cell walls. The bar indicates mean ± SE (*n* = 10). (**C**) Lignin content. (**D**) POD activities. (**E**) PAL activities. The bar indicates mean ± SE (*n* = 3). ** and * indicate significant differences at *p *< 0.01 and *p* < 0.05 between two treatments, respectively, and ns indicates no significant difference between treatments. Different letters above the bars indicate significant differences at *p *< 0.05 level among different treatments. ANOVAs of Cd, P, and their interaction (Cd * P) are indicated (*, *p* < 0.05; **, *p* < 0.01; ns, not significant). SP: sufficient P, DP: deficient P.

Furthermore, we observed the development of SL (Figure 2). The results showed that Cd exposure significantly accelerated SL deposition, regardless of the P treatments. Deficient P significantly induced SL development under Cd exposure, but its effect did not appear to be significant under 0 Cd conditions. In all treatments, the latest-formed SL was recorded in the combinations of 0 Cd and sufficient P conditions, with fluorescent signals indicating SL formation at a distance of 10.0% from tips. On the other hand, the earliest-formed SL was observed in the combination of Cd exposure and P-deficiency conditions, at a distance of 5% from tips, while fully developed SL was observed at 30% from the root tips. In addition, TEM results revealed a significant increase in endodermal cell wall thickness, approximately 37.2% thicker under Cd exposure in P-deficiency conditions compared to P-sufficiency conditions (Figure 3A,B).

### 2.2. P Deficiency Affects the Activities of Key Enzymes Related to Apoplastic Barrier Formation and the Levels of Lignin

We determined root lignin content and examined the activities of POD and PAL enzymes associated with lignin and suberin biosynthesis. The findings indicated a significant increase in root lignin content under P-deficiency conditions, regardless of Cd conditions (Figure 3C). In addition, both P deficiency and Cd exposure led to elevated root POD activities. Specifically, under Cd stress, POD activities increased by 24.6% under deficient P conditions compared to sufficient P (Figure 3D). In the absence of Cd, PAL activities did not exhibit significant differences at both P levels. However, a significant increase in PAL activities was observed at deficient P under Cd exposure (Figure 3E).

### 2.3. P Deficiency Affects Net Cd^2+^ Influx in Root Tips Under Cd Exposure

**Figure 4:**
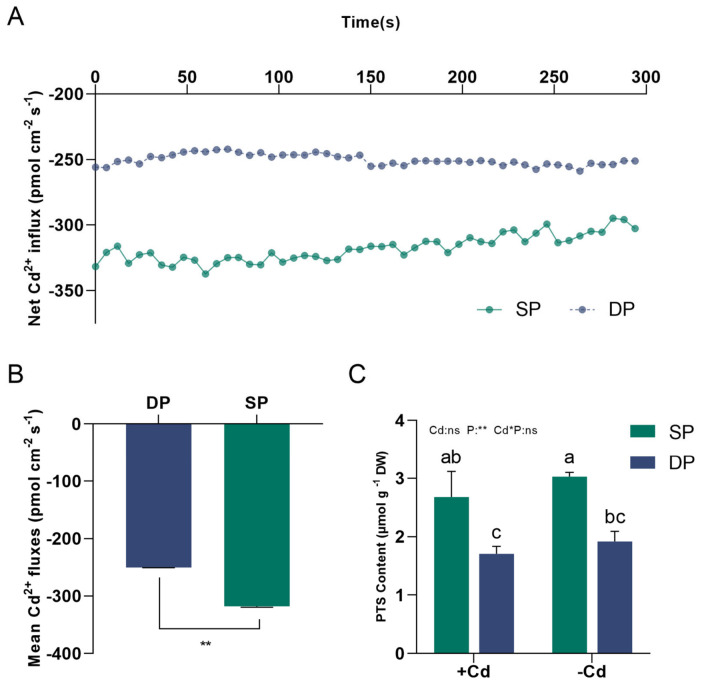
Effect of P deficiency on net Cd^2+^ influx at approximately 1.5% from the root tip under Cd stress, and the effects of P and Cd treatments on 8-hydroxy-1,3,6-pyrenetrisulfonic acid trisodium salt (PTS) content in leaves. (**A**) Transient Cd^2+^ fluxes. (**B**) Mean Cd^2+^ fluxes. Positive values represent efflux and negative values represent influx. (**C**) PTS contents in leaves. The bar indicates mean ± SE (*n* = 3). Different letters above the bars indicate significant differences at *p *< 0.05 among different treatments. ** and * indicate significant differences at *p *< 0.01 and *p* < 0.05 between two treatments, respectively, and ns indicates no significant difference between treatments. ANOVAs of Cd, P, and their interaction (Cd * P) are indicated (*, *p* < 0.05; **, *p* < 0.01; ns, not significant). SP: sufficient P, DP: deficient P.

We determined the Cd^2+^ influx at a distance of 1.5% from root tips under 50 μmol Cd exposure (Figure 4A). The results indicated that the Cd^2+^ influx remained relatively constant throughout the duration of the measurements. The effect of P on Cd^2+^ influx was consistent with what we observed in the development of CS. Under Cd stress, the mean value of Cd^2+^ influx in root tips was −250.3 pmol cm^−2^ s^1^ under P-deficiency conditions and −317.9 pmol cm^−2^ s^−1^ under P-sufficiency conditions. The mean Cd^2+^ influx was reduced by 21.3% under deficient P compared to sufficient P (Figure 4B).

### 2.4. P Deficiency Affects the Translocation of PTS in Apoplastic Pathway

PTS was used to verify whether the P and Cd treatments had an effect on transport in the apoplastic pathway (Figure 4C). The results indicated a decreasing trend in leaf PTS content under Cd exposure, although the changes were not statistically significant. In addition, P had a notably influence on the apoplastic transport. Deficient P resulted in a reduction in PTS concentration in leaves, whether or not Cd treatment was applied. Specifically, under Cd stress, the PTS content in leaves reduced by 36.3% under P-deficiency conditions compared to that under P-sufficiency conditions.

### 2.5. Cd Accumulation Aboveground

**Figure 5:**
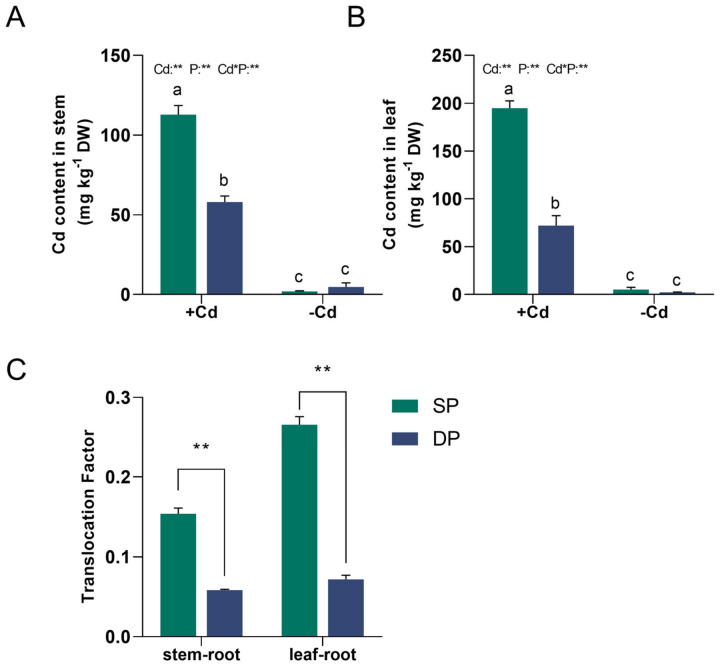
Cd content in stems (**A**), leaves (**B**) and TF of Cd between different organs under Cd exposure (**C**). The bar indicates mean ± SE (*n* = 3). Different letters above the bars indicate significant differences at *p* < 0.05 among different treatments. ** and * indicate significant differences at *p* < 0.01 and *p* < 0.05 between two treatments, respectively, and ns indicates no significant difference between treatments. ANOVAs of Cd, P, and their interaction (Cd * P) are indicated (*, *p* < 0.05; **, *p* < 0.01; ns, not significant). SP: sufficient P, DP: deficient P.

The content of Cd in *Salix caprea* was significantly increased under Cd exposure. Furthermore, it was observed that sufficient P increased the Cd content in stems and leaves, with an increase of 94.7% in stems and 170.5% in leaves compared to deficient P conditions (Figure 5A,B). The results of TF of Cd showed that sufficient P significantly promoted the translocation of Cd to aboveground tissues (Figure 5C).

### 2.6. Identification of Key Genes Regulated by P in Apoplastic Barrier Formation Under Cd Stress

Transcriptome sequencing was performed among different P levels under Cd stress. The result of the principal component analysis showed that PC1 and PC2 cumulatively explained 93.3% of the total sample variance (76.5% and 16.8% for PC1 and PC2, respectively) and were significantly different between treatments (Figure S1A). In addition, the inter-sample correlation analysis revealed a high degree of reproducibility for the three replicates within the group (Figure S1B).

**Figure 6:**
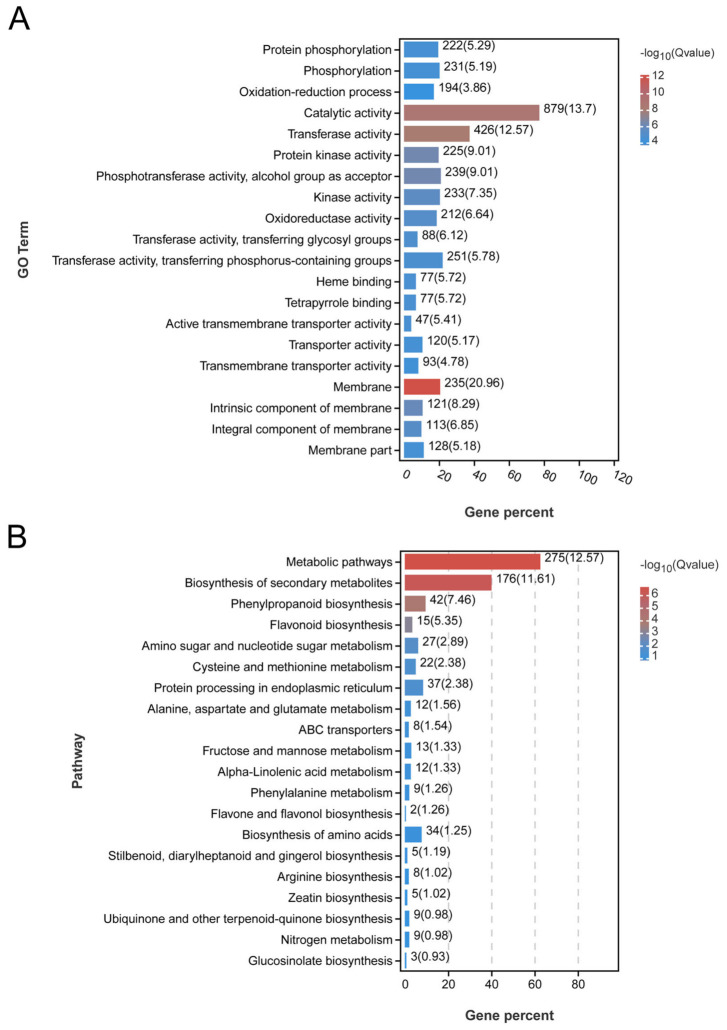
Enrichment analysis of differentially expressed genes. (**A**) GO enrichment analysis. (**B**) KEGG enrichment analysis.

We screened with *p*-adjust < 0.05 and |log2FC| ≥ 2, and obtained a total of 2596 differential genes, of which 1082 were upregulated and 1514 were downregulated (Figure S1C). GO and KEGG enrichment analyses were performed on the screened genes. In GO analysis, membrane (GO:0016020), catalytic activity (GO:0003824), and transferase activity (GO:0016740) were at the forefront of the most significant enrichment (Figure 6A). KEGG enrichment analysis showed that the pathways that were significantly enriched between the two groups included metabolic pathways, biosynthesis of secondary metabolites, and phenylalanine biosynthesis (Figure 6B).

**Figure 7:**
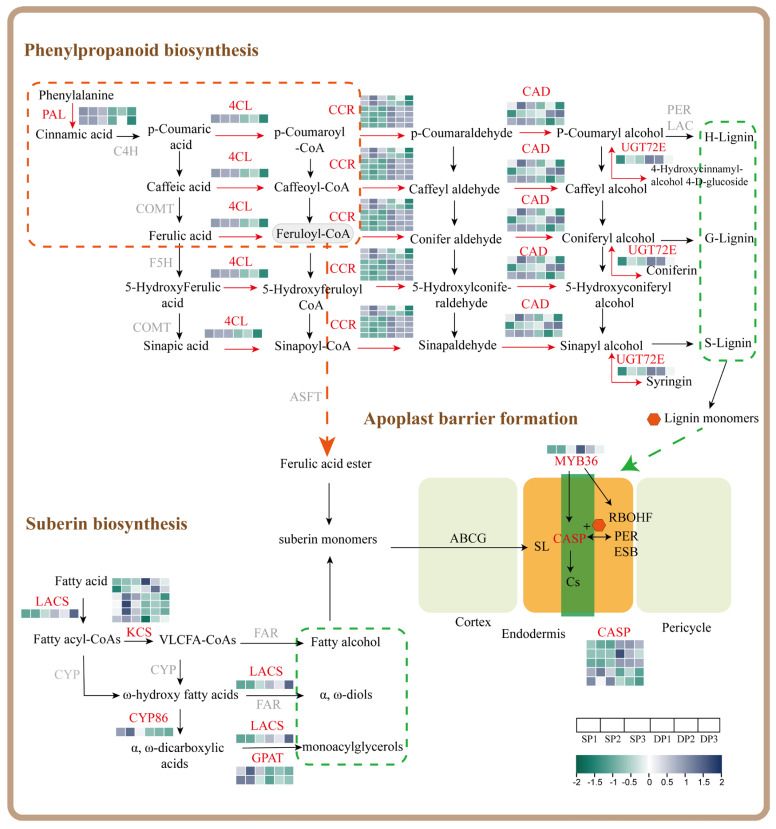
Schematic diagram of the regulatory network for apoplastic barrier formation, showing the polymerization of lignin, suberin monomers, and apoplastic barriers in the root endodermis. Red-colored genes indicate differentially expressed genes in the pathway. The heatmap represents gene expression levels. SP: sufficient P; DP: deficient P.

A total of 30 differentially expressed genes (DEGs) related to apoplastic barrier formation were identified (Figure 7, Table S2). For lignin monomer synthesis, 13 DEGs were identified, including two phenylalanine ammonia-lyase genes (*PAL*, two downregulated), one 4-coumarate-CoA ligase gene (*4CL*, one downregulated), six cinnamoyl-CoA reductase genes (*CCR*, two downregulated and four upregulated), three cinnamyl alcohol dehydrogenase genes (*CAD*, two downregulated and one upregulated), and one UDP-glucosyltransferase gene (*UGT72E1*, one upregulated). For suberin monomer synthesis, a total of 11 DEGs were identified, including one long-chain acyl-CoA synthetase gene (*LACS*, one upregulated), six β-ketoacyl-CoA synthase genes (*KCS*, four downregulated and two upregulated), one cytochrome P450 family 86 protein gene (*CYP86*, one upregulated), and two glycerol-3-phosphate acyltransferase genes (*GPAT*, two downregulated). For the establishment of casparian strips, a total of six DEGs were identified, including five casparian strip membrane domain protein genes (*CASP*, two downregulated and three upregulated) and one MYB domain protein 36 gene (*MYB36*, one upregulated).

## 3. Discussion

### 3.1. P-Deficiency-Mediated Development of the Apoplastic Barriers Inhibits Radial Transport of Cd

The functional integrity of the endodermis, serving as an important selective barrier for material transport in roots, relies on the differentiation of the endodermis to form the apoplastic barriers, the CS and SL [5,30]. Recent studies highlighted the plasticity of apoplastic barriers in the adaptive response of plants to various abiotic stresses, such as heavy metal stress, drought stress, and salt stress [18,31,32,33,34]. As a representative nonessential heavy metal, Cd has been extensively studied for its role in apoplastic barrier formation. For example, in Sedum alfredii, Cd significantly induced the development of CSs and SL [35]. Similar apoplastic barrier development in response to Cd stress has been observed in rice [36], wheat [4], maize [37], and willow [38]. In our study, Cd exposure accelerated the deposition of CS and SL, regardless of P levels (Figure 1 and Figure 2). This suggests that the accelerated deposition of apoplastic barriers may be an effective evolutionary strategy for plants to prevent toxic heavy metals from entering the xylem. In addition, P deficiency also promoted the development of apoplastic barriers and accelerated their establishment under Cd stress (Figure 1 and Figure 2). This could be attributed to the heightened plasticity of apoplastic barriers under P-deficiency conditions. Previous studies have demonstrated the involvement of various mineral elements in regulating apoplastic barrier development to maintain plant mineral homeostasis. For the CS, low N and low P were observed to favor CS development [28]. For SL, low N, low P, and deficiencies of S and K were demonstrated to promote SL formation, while deficiencies of Fe, Mn, and Zn inhibited SL establishment [28,39,40]. In summary, we hypothesize that deficiencies in macro elements may facilitate apoplastic barrier development, while deficiencies in trace elements may inhibit it, possibly influenced by the element’s availability and plant demand. Additionally, our results indicated that deficient P led to a notable decrease in the contents of K and S, along with a remarkable increase in Cd content in roots under Cd exposure (Figure S2). This intensified the stress in both Cd and nutrient conditions. The disruption of plant ion uptake due to deficient P under Cd exposure could be a significant factor accelerating the induction of apoplastic barrier development. The contrasting effects of Cd stress and P deficiency in inducing apoplastic barriers highlight the crucial property of the endodermis as a bi-directional barrier, preventing not only the entry of substances into the xylem but also the leakage of accumulated ions from the stele [41].

Although numerous investigations have demonstrated the important role of apoplastic barriers in regulating water and ion transport [42], the impact of P-deficiency-induced apoplastic barriers under Cd exposure on apoplastic transport remains unclear. PTS, a widely used apoplastic bypass tracer, is valuable for assessing apoplastic pathways as it exclusively relies on apoplastic transport [43]. For example, in Elymus sibiricu, PTS was used to demonstrate the limiting effect of the drought-induced apoplastic barriers on apoplastic transport [44]. In our study, the lower leaf PTS content under P-deficiency conditions consistently aligned with an earlier developed apoplastic barrier under P-deficiency conditions, indicating significant regulation of apoplastic transport by apoplastic barriers in *Salix caprea* roots (Figure 1 and Figure 2 and Figure 4C). In addition, although the apoplastic pathway is not typically considered a primary pathway for Cd transport, emerging evidence underscores the importance of root tips unaffected by apoplastic barriers for Cd uptake. In both Populus and sedum, Cd^2+^ influx exhibited a downward decreasing trend with an increasing distance from root tips [45,46]. In our study, deficient P notably decreased Cd^2+^ influx at root tips, indicating that P-deficiency-induced CS deposition under Cd stress significantly inhibited Cd uptake (Figure 4A,B). Therefore, although the contribution of the apoplastic pathway to total Cd translocation remains uncertain, we can conclude that the development of apoplastic barriers induced by P deficiency is a key factor responsible for the reduction in aboveground Cd enrichment under P-deficiency conditions (Figure 5). Based on the above results, we speculate that sufficient P could facilitate Cd translocation to the shoots of *Salix caprea* when used for the remediation of mining areas or Cd-contaminated soils, thereby enhancing remediation efficiency. It should be emphasized, however, that hydroponic conditions cannot fully replicate the complexity of soil environments. Accordingly, further validation is required before extending these findings to agricultural soils or other field scenarios.

### 3.2. Physiological and Molecular Mechanisms Involved in P-Deficiency-Induced Development of Apoplastic Barriers Under Cd Stress

The formation of apoplastic barriers depends on the deposition of two impermeable polymers, lignin and suberin [5]. Consequently, the biosynthesis of lignin and suberin monomers and their polymerization in cell walls determine the developmental process of apoplastic barriers. Despite the controversy, it is now generally recognized that lignin is the primary constituent of CS [30,47,48,49]. Our results indicate that deficient P markedly reduced lignin content in roots under Cd exposure, which is consistent with the observed CS development (Figure 1 and Figure 2 and Figure 3C). This suggests a significant positive correlation between lignin content and CS development, a relationship also demonstrated in species such as sedum and rice [36,50]. Lignin monomers originate from phenylalanine and are synthesized intracellularly through a series of hydroxylation, methylation and reduction reactions [51,52]. PAL, CCR and POD have been shown to play crucial roles in the biosynthesis of lignin monomers as initiating enzymes in the phenylalanine pathway, initiating enzymes in the lignin-specific synthesis pathway and lignin polymerases, respectively. For example, overexpression of *PAL* and *CCR* genes increased lignin content in Ricinus and Populus, respectively, while decreased lignin content was observed in POD-silenced Manihot [53,54,55]. Our results showed that under Cd stress, PAL and POD enzyme activities, as well as genes encoding CCR enzymes, were significantly upregulated under P-deficiency conditions, which may explain the increase in lignin content under P deficiency (Figure 3 and Figure 7). Suberin is a glycerol-based polymer of polyolipids and polyphenols [56]. *LACS* and *KCS* genes have been observed to play important roles in the biosynthesis of suberin [57]. LACS, a crucial enzyme in fatty acid metabolism, activates fatty acids to fatty acyl-coenzyme A thioesters, initiating the synthesis of suberin monomers from long-chain fatty acids [58]. Additionally, KCS is a rate-limiting enzyme involved in very-long-chain fatty acid (VLCFA) synthesis, an important precursor for suberin monomers. Overexpression of the *LACS* and *KCS* genes in Arabidopsis mutants has been shown to facilitate suberin biosynthesis [59,60]. In our study, P deficiency promoted the relative expression of *LACS* and *KCS* genes under Cd stress (Figure 7). Therefore, it is reasonable to suggest that *LACS* and *KCS* are crucial genes mediating P-deficiency-induced suberin biosynthesis, which promotes the production of suberin monomers by accelerating fatty acid activation and VLCFA synthesis.

Recently, systematic progress has been achieved in characterizing the molecular mechanisms of CS deposition in the cell wall, with particular attention given to the significant role of the CASP gene and the transcription factor gene *MYB36*. *CASP* genes, membrane proteins precisely localized in the region of CS formation, are responsible for the initial polymer backbone formation in the CS assembly process [61]. *CASP* genes accurately regulate the polymerization process of lignin through synergistic interactions with other genes, such as respiratory burst oxidase homolog F (*RBOHF*), enhanced suberin 1 (*ESB1*), etc. [47,62]. The regulatory role of *CASP* genes in CS establishment has been demonstrated in various species [63,64]. Similarly, we observed that under Cd stress, the *CASP* gene was significantly upregulated under P-deficiency conditions, suggesting its pivotal role in casparian strip formation induced by P deficiency (Figure 7). *MYB36* stands out as one of the most crucial transcription factors in CS deposition, playing a pivotal role in regulating the expression of key genes linked to CS formation, such as *CASP*, *POD*, and *ESB* [65,66]. Thus, the consistent expression patterns of *MYB36* and *CASP* in this study imply that deficient P may contribute to CS formation under Cd stress by regulating the *MYB36* genes and downstream target genes associated with CS establishment (Figure 7). In conclusion, we hypothesize that P deficiency plays two major roles in promoting CS and SL formation under Cd stress. Firstly, it enhances the accumulation of lignin and suberin monomers by upregulating the relative expression of genes such as *CCR*, *CAD*, *LACS*, and *KCS*. Secondly, it facilitates the precise localization of the CS by inducing the expression of the *MYB36* transcription factor and *CASP* genes. It is important to note that the identification of key genes in this study was performed under Cd stress to more closely reflect natural environmental conditions. The individual and interactive effects of P and Cd on the expression of these genes remain to be elucidated in future studies. Additionally, our previous study found that the expression levels of genes encoding the *HMA*, *ZIP*, *NRAMP*, *CAX*, and *ABC* transporter families were positively regulated by P levels [67]. In addition, the regulatory effects of other mineral elements, such as K, on Cd-related transport proteins have also been documented, indicating that mineral element-mediated regulation of Cd transporters is another key factor influencing Cd translocation [68]. Therefore, although the present study primarily focused on the regulation of apoplastic Cd transport by P, it is reasonable to suggest that Cd transporter proteins also play a crucial role in limited Cd translocation under deficient P conditions.

## 4. Materials and Methods

### 4.1. Plant Cultivation and Treatment

A *Salix caprea* clone, characterized by its high enrichment to Cd, was used as the experimental material for this study. This ecotype was collected from the Pb/Zn mining area in Ruyang County, Henan Province, China. The clone of this ecotype was obtained through cuttings and cultivated for four months. After four months of greenhouse culture, the plants were pre-cultured hydroponically in a modified 1/2 Hoagland nutrient solution (NSP1020, Coolaber, Beijing, China) for three weeks. Plants with similar heights, approximately 60 cm, were selected for the experiment. The experimental involved two Cd levels (−Cd: 0 µmol L^−1^ and +Cd: 50 µmol L^−1^) and two P levels (sufficient P, SP: 0.5 mmol L^−1^ and deficient P, DP: 0.01 mmol L^−1^). To eliminate the effects of K deficiency due to KH_2_PO_4_ deficiency, 0.245 mmol L^−1^ of K_2_SO_4_ was added to the deficient P solution. The detailed concentrations of the elements are shown in Table S1. Each treatment consisted of three replicates, with each replicate represented by an independent hydroponic container (8 L). Four plants were placed in each container, and the average value was used as the data for each replicate. The nutrient solution was adjusted to pH 5.5 and renewed every three days until harvest. The treatment solution was replaced every three days. Each group of treatments contained 12 plants and all treatments lasted for 28 days.

### 4.2. Determination of Element Concentrations

The dried plant samples were ground into a homogeneous powder. Subsequently, 0.2 g of sample was weighed into a digestion tube, soaked overnight in HNO_3_, and digested at 160 °C for 6 h. Twelve elements, including P, Cd, potassium (K), sodium (Na), sulfur (S), copper (Cu), aluminum (Al), magnesium (Mg), zinc (Zn), Ca, iron (Fe), and manganese (Mn), were measured using an inductively coupled plasma atomic emission spectrometer (Agilent 710 ICP-OES, Agilent Technologies, Santa Clara, CA, USA). The translocation factor (TF) of Cd was calculated according to the Cd concentrations.

### 4.3. Histochemical Observation of Apoplastic Barriers

After 28 days of treatment, roots of about 10 cm in length were selected, embedded in agarose (5% *w*/*v*) and then cut into 30 μm-thick transverse sections (slices per 1 mm at 0–10 mm, per 10 mm at 10–50 mm) using a vibratome.

For the observation of the CS, the sections were immersed in a solution of 0.2% Berberine Hemisulfate (*w*/*v*, dissolved in lactic acid), and stained in the dark at 70 °C for 1 h. The sections were then washed in distilled water and restained with 0.1% toluidine blue for 20 min, and then washed three times before being observed.

For observation of the SL, sections were stained by immersion in a solution of 0.2% Fluorol Yellow 088 (*w*/*v*, dissolved in lactic acid) stained in the dark at 70 °C for 1 h. Sections were then washed in distilled water and restained with 0.1% toluidine blue for 20 min, and then washed three times before being observed.

After staining, cross-sections were visualized and photographed using a fluorescence microscope (Eclipse 80i, Nikon Corporation, Tokyo, Japan). CS was observed at excitation and emission wavelengths of 405 nm and 450–550 nm, respectively, while SL was observed at excitation and emission wavelengths of 488 nm and 485–545 nm, respectively. For quantification, the distance from the root tip to the first section of CS and SL formation was documented, and the percentage of the distance to the whole root length was calculated.

### 4.4. Transmission Electron Microscope (TEM) Observation Root

Root segments, located 1–2 cm from the root tip, were fixed overnight in a 2.5% (*v*/*v*) glutaraldehyde solution prepared in 0.1M phosphate buffer solution (pH 7.0). Afterward, the samples underwent a 7 h fixation in 1% osmium tetroxide dissolved in PBS and were subsequently dehydrated through a series of graded ethanol and acetone. Following dehydration, the specimens were embedded in resin and cross-sectioned using an ultramicrotome (Leica UC7, Leica, Düsseldorf, Germany). Photomicrographs were then taken and analyzed using TEM (Hitachi HT7800, Hitachi High-Tech Corporation, Tokyo, Japan).

### 4.5. Determination of Lignin and Key Enzyme Activities

Lignin content and phenylalanine ammonia-lyase (PAL) and peroxidase (POD) activities were detected using kits (BC4200, BC0210, BC0095, Solarbio, Beijing, China) following the manufacturer’s protocol, respectively.

### 4.6. Measurement of Net Cd^2+^ Fluxes

The non-invasive micro-test technology (NMT) was employed to record the net fluxes of Cd^2+^. After 28 days of treatment, primary roots from different treated plants were selected and fixed in a measuring solution, and equilibrated for 20 min. Afterward, the roots were transferred to a fresh measuring solution, and the steady-state Cd^2+^ fluxes at a distance of 1500 μm from the root tips were determined and recorded for 300s.

### 4.7. Trisodium-8-hydroxy-1,3,6-pyrenetrisulphonic Acid (PTS) Analyses

To investigate whether P and Cd had an effect on the transport through apoplastic pathway, some plants were cultured for an additional 5 days with a treatment solution supplemented with PTS (a nontoxic and widely used tracer to show the pathway of apoplastic transport). The leaves were collected, and the dried sample powder was transferred into 10 mL of distilled water, then maintained at 90 °C for 2 h. PTS fluorescence was measured using fluorescence spectrophotometry (Varioskan LUX, Thermo Fisher Scientific, Waltham, MA, USA). The excitation and emission wavelengths were 380 nm and 510 nm, respectively.

### 4.8. Transcriptome Analysis

Total RNA was extracted using the Trizol Reagent Kit (Invitrogen, Carlsbad, CA, USA) according to the manufacturer’s protocol. The cDNA library was constructed and subsequently sequenced using the Illumina NovaSeq6000 platform (Illumina Inc., San Diego, CA, USA). Raw sequencing data (raw reads) were filtered to remove low-quality sequences and junctions, resulting in high-quality sequencing data (clean reads). The high-quality sequencing data were aligned to the *Salix purpurea* reference genome (V5.1, https://phytozome-next.jgi.doe.gov/info/Spurpurea_v5_1, accessed on 1 July 2025) to annotate the function of genes. RNA differential expression analysis was performed between two groups using the DESeq2 package. Genes with a false discovery rate (FDR) < 0.05 and an absolute fold change ≥ 2 were considered differentially expressed (DEGs). Differentially expressed genes (DEGs) were identified through significant difference analysis (*p* < 0.05) and annotated in the Gene Ontology (GO) or Kyoto Encyclopedia of Genes and Genomes (KEGG) databases.

### 4.9. Statistical Analysis

The normality of the data was tested prior to statistical analyses using the Shapiro–Wilk test. To assess the effects of P and Cd treatments, two-way analysis of variance (ANOVA) was performed to evaluate the main effects of Cd, P, and their interaction on each variable. For single-factor comparisons involving more than two groups, one-way ANOVA was applied, followed by Duncan’s multiple range test to identify significant differences (*p* < 0.05), which are indicated by different letters. For pairwise comparisons, independent-sample t-tests were conducted, with * and ** denoting significance at *p* < 0.05 and *p* < 0.01, respectively. Data are presented as mean ± standard error (SE). Statistical analyses and graphing were performed using GraphPad Prism 8.

## 5. Conclusions

Different levels of P supply resulted in differential Cd translocation in *Salix caprea*, which was partly attributed to the degree of development of P-induced apoplastic barriers. Under Cd stress, P deficiency led to a significant upregulation of genes associated with lignin and suberin monomer biosynthesis (*CCR*, *CAD*, *LACS*, and *KCS*), as well as genes related to casparian strip localization (*MYB36* and *CASP*), resulting in earlier formation of apoplastic barriers. This reduced Cd translocation to aboveground tissues via the apoplastic pathway. These findings elucidate the regulatory mechanism of P deficiency in Cd translocation via the apoplastic pathway and provide new insights into the rational use of P fertilizers in phytoremediation.

## Data Availability

The original contributions presented in this study are included in the article/Supplementary Materials. Further inquiries can be directed to the corresponding author.

## Supplementary Materials

The following supporting information can be downloaded at: https://www.mdpi.com/article/10.3390/plants15111728/s1, Figure S1: Transcriptome analysis of P-treated *Salix caprea* root systems under Cd stress. (A) PCA of 6 samples. (B) Correlation analysis of 6 samples. (C) Volcanic map of differential genes. Figure S2: Hierarchical cluster analysis (HCA, a) and Spearman’s correlation analysis (b) for the root elements. Solid lines represent R > 0.7 or <−0.7 and *p* < 0.05, whereas dashed lines represent R > 0.7 or <−0.7 and *p* > 0.05. Red lines denote positive correlations; blue lines denote negative correlations; Table S1: The detailed element concentration under different P treatments. Table S2: Differential genes associated with apoplastic barrier formation.
