# Supplementary material for: Phosphorus-Deficiency-Induced Development of Root Apoplastic Barriers Restricts Cadmium Translocation in Salix caprea
Source: Plants (Basel). 2026 Jun 3;15(11):1728. doi: 10.3390/plants15111728 (PMC13259451; doi:10.3390/plants15111728)
Supplement: Supplementary file 1 [file plants-15-01728-s001.zip › plants-4335432-supplementary.pdf]

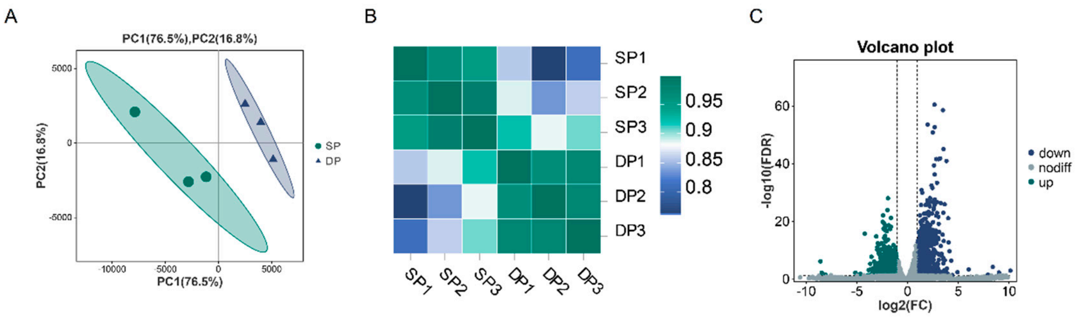

Fig. S1. Transcriptome analysis of P-treated *Salix caprea* root systems under Cd stress. (A) PCA of 6 samples. (B) Correlation analysis of 6 samples. (C) Volcanic map of differential genes.

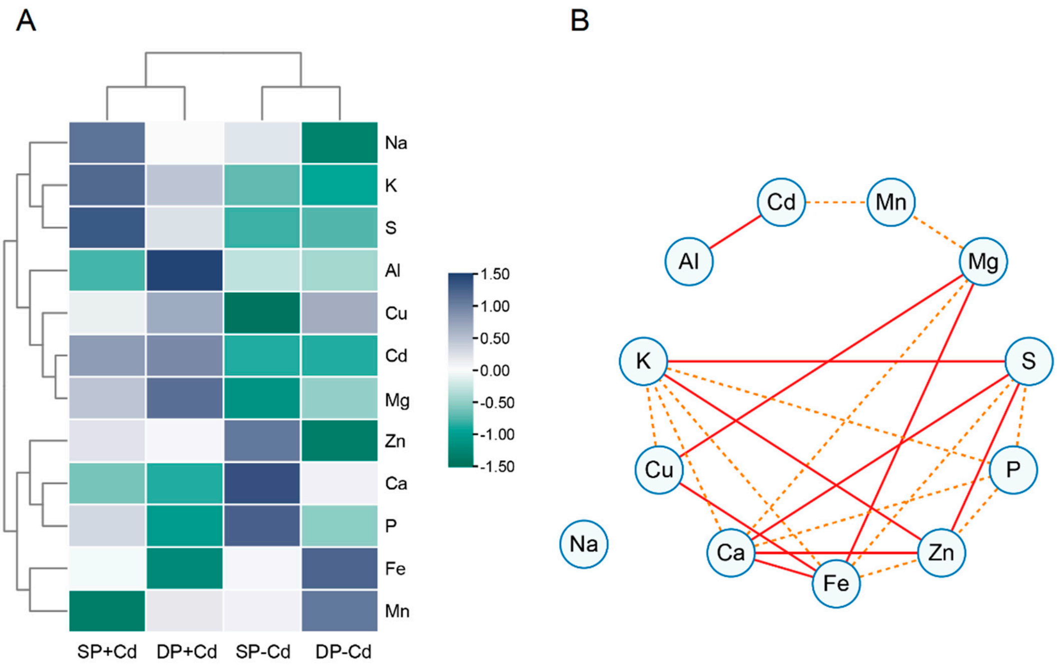

Fig. S2. Hierarchical cluster analysis (HCA, a) and Spearman's correlation analysis (b) for the root elements. Solid lines represent $R>0.7$ or <-0.7 and $P<0.05$, whereas dashed lines represent $R>0.7$ or <-0.7 and $P>0.05$. Red lines denote positive correlations, blue lines denote negative correlations.

Supplementary Table. S1. The detailed element concentration under different P treatments

Macronutrient	***Final concentration (mM)***	
	Sufficient P solution	***Deficient P solution***
Compound		
$Ca(NO_3)_2 \cdot 4H_2O$	2	2
KNO_3	3	3
NH_4NO_3	0.25	0.25
$(NH_4)_2SO_4$	0.5	0.5
$MgSO_4$	1	1
KH_2PO_4	0.5	0.01
Fe-EDTA	0.05	0.05
KI	0.0025	0.0025
H_3BO_3	0.05	0.05
$MnSO_4 \cdot H_2O$	0.05	0.05
$ZnSO_4 \cdot 7H_2O$	0.015	0.015
$Na_2MoO_4 \cdot 2H_2O$	0.0005	0.0005
$CuSO_4 \cdot 5H_2O$	0.00005	0.00005
$CoCl_2 \cdot 6H_2O$	0.00005	0.00005
K_2SO_4	0	0.245
Nutrient/Ion		
Ca^{2+}	2	2
NO_3^-	7	7
K^+	3.5025	3.5025
NH_4^+	1.25	1.25
Mg^{2+}	1	1
SO_4^{2-}	1.565	1.81
HPO_4^{2-}	0.5	0.01
Fe^{2+}	0.05	0.01
I^-	0.0025	0.0025
BO_3^{3-}	0.05	0.05
Mn^{2+}	0.05	0.05
Zn^{2+}	0.015	0.015
Cl^-	0.0001	0.0001
Na^+	0.001	0.001
Cu^{2+}	0.00005	0.00005
Co^{2+}	0.00005	0.00005
Mo^{6+}	0.0005	0.0005

Supplementary Table. S2. Differential genes associated with apoplastic barriers formation

Gene ID	FPKM SP1	FPKM SP2	FPKM SP3	FPKM DP1	FPKM DP2	FPKM DP3	Symbol	log2 (fc)	FDR
Sapur.010G179400	11.56	10.15	8.32	1.12	1.81	0.67	*PAL1*	-3.06	0.00
Sapur.008G028000	22.28	22.94	17.43	6.18	12.83	4.3	*PAL1*	-1.43	0.00
Sapur.019G055800	236.28	219.91	218.64	109.7	122.95	79.13	*4CL3*	-1.11	0.00
Sapur.001G036900	9.37	13.75	8.55	3.73	6.14	1.96	*CCR*	-1.42	0.01
Sapur.003G137800	137.38	184.41	153.89	77.01	72.24	53.83	*CCR*	-1.23	0.00
Sapur.001G035200	6.17	7.4	4.85	43.77	28.88	34.93	*CCR*	2.55	0.00
Sapur.001G035100	99.42	76.45	77.28	643.71	409.92	499.48	*CCR*	2.62	0.00
Sapur.001G035300	8.01	5.22	8.61	52.39	46.5	42	*CCR*	2.69	0.00
Sapur.001G037000	11.6	9.78	10.46	102.58	73.2	93.38	*CCR*	3.08	0.00
Sapur.002G012800	9.62	18.61	13.49	4.85	7.67	3.16	*CAD9*	-1.41	0.00
Sapur.009G048900	3.47	5.47	6.15	6.71	12.76	14.24	*CAD7*	1.16	0.00
Sapur.016G154800	86.2	95.14	88.73	44.41	31.95	46.16	*CAD7*	-1.14	0.00
Sapur.007G027000	0.64	1.72	1.43	4.26	3.96	2.06	*UGT72E1*	1.44	0.01
Sapur.008G069900	2.43	1.63	1.47	8.26	8.64	7.03	*CASP*	2.11	0.00
Sapur.012G019800	0	0	0	0.98	0.53	0.11	*CASP*	9.08	0.01
Sapur.013G121800	1.7	1.59	2.12	6.24	6.13	5.69	*CASP*	1.74	0.00
Sapur.016G286900	17.69	23.17	14.57	5.48	8.31	5.35	*CASP*	-1.53	0.00
Sapur.010G164100	4.73	2.92	4.81	2.15	1.73	1.33	*CASP*	-1.26	0.04
Sapur.004G162300	1.87	1.86	2.96	6.26	4.28	3.01	*MYB36*	1.02	0.02
Sapur.013G022400	9.31	9.55	13.53	23.37	19.07	35	*ATLACS7*	1.26	0.00
Sapur.010G168900	0	0.04	0.08	0.9	0.43	0.2	*KCS19*	3.67	0.01
Sapur.013G013900	0.44	1.17	1.67	2.31	3.79	2.35	*KCS7*	1.37	0.01
Sapur.008G129900	21.78	38.52	30.77	14.38	14.83	9.66	*KCS2*	-1.22	0.00
Sapur.002G139400	2.38	6.82	2.74	1	1.33	1.05	*KCS1*	-1.82	0.00
Sapur.010G067200	5.6	13.22	7.28	2.87	3.32	2.53	*KCS11*	-1.58	0.00
Sapur.018G026500	117.48	148.04	121.2	24.13	58.56	72.86	*KCS11*	-1.31	0.01
Sapur.008G045700	11.89	21.49	12.72	4.78	5.83	4.91	*ATGPAT5*	-1.57	0.00
Sapur.005G159100	1.98	1.97	0.76	0.28	0.65	0.52	*GPAT1*	-1.70	0.04
Sapur.014G066200	0.72	0.92	0.39	0.1	0.06	0.02	*CYP86*	-3.50	0.00
